# Biopsy-Controlled Liver Fibrosis Staging Using the Enhanced Liver Fibrosis (ELF) Score Compared to Transient Elastography

**DOI:** 10.1371/journal.pone.0051906

**Published:** 2012-12-19

**Authors:** Kristin Wahl, William Rosenberg, Bernhard Vaske, Michael P. Manns, Klaus Schulze-Osthoff, Matthias J. Bahr, Heike Bantel

**Affiliations:** 1 Department of Gastroenterology, Hepatology and Endocrinology, Hannover Medical School, Hannover, Germany; 2 Centre for Hepatology, University College London Institute of Liver and Digestive Health, Division of Medicine, University College London, London, United Kingdom; 3 Institute of Biometry, Hannover Medical School, Hannover, Germany; 4 Interfaculty Institute of Biochemistry, University of Tübingen, Tübingen, Germany; 5 Sana Kliniken, Lübeck, Germany; University of Navarra School of Medicine and Center for Applied Medical Research (CIMA), Spain

## Abstract

**Background and Aims:**

Chronic liver diseases are characterized by inflammatory and fibrotic liver injuries that often result in liver cirrhosis with its associated complications such as portal hypertension and hepatocellular carcinoma. Liver biopsy still represents the reference standard for fibrosis staging, although transient elastography is increasingly used for non-invasive monitoring of fibrosis progression. However, this method is not generally available and is associated with technical limitations emphasizing the need for serological biomarkers staging of liver fibrosis. The enhanced liver fibrosis (ELF) score was shown to accurately predict significant liver fibrosis in different liver diseases, although extracellular matrix components detected by this score may not only mirror the extent of liver fibrosis but also inflammatory processes.

**Methods:**

In this prospective biopsy-controlled study we evaluated the utility of the ELF score in comparison to transient elastography to predict different stages of fibrosis in 102 patients with chronic liver diseases.

**Results:**

Both techniques revealed similar area under receiver operating characteristic curve values for prediction of advanced fibrosis stages. Compared to transient elastography, the ELF score showed a broader overlap between low and moderate fibrosis stages and a stronger correlation with inflammatory liver injury.

**Conclusions:**

Both the ELF score as well as transient elastography allowed for high quality fibrosis staging. However, the ELF score was less discriminative in low and moderate fibrosis stages and appeared more strongly influenced by inflammatory liver injury. This should be considered when making clinical interpretations on the basis of ELF score values.

## Introduction

Liver fibrosis is the consequence of a variety of chronic liver diseases and can result in liver cirrhosis. Early detection of fibrosis progression and development of cirrhosis are crucial for management of patients with chronic liver diseases since advanced fibrosis is associated with clinical complications and formation of hepatocellular carcinoma. Although liver biopsy remains the reference standard for evaluating liver fibrosis, it is limited by sampling errors and risk of complications [1], [2]. In addition to sampling errors, intra- and interobserver variability may lead to misinterpretation of the fibrosis stage [3]–[6]. One reason for the difficulties in correctly assessing the fibrosis stages might base on biopsy specimen that only represents 1/50.000 of the total liver mass [1].

The liver volume explored by transient elastography is estimated to be 100 times larger compared to liver biopsy and might thus be more representative of the entire organ [7]. Fibrosis is a dynamic process and monitoring of fibrosis is desirable to obtain information not only about disease progression but also about treatment efficacy. Much attention has therefore been focused on the development of non-invasive methods to detect liver fibrosis. Measurement of liver stiffness by transient elastography is a widely accepted method for non-invasive liver disease staging. However, this technique is cost-intensive and its availability is largely limited to liver centers. Moreover, liver stiffness measurements can be difficult or impossible in obese patients, in those with narrow intercostal space or in patients with ascites [8] and a failure rate up to 18.9% has been reported following a review of 13,369 examinations over a 5-year period [9]. Substantial effort has been devoted to develop routine laboratory tests for fibrosis assessment, including the FibroTest®, Hepascore® and markers of extracellular matrix components or enzymes involved in their degradation or synthesis [10]–[15]. The combination of some of those parameters, such as hyaluronic acid (HA), tissue inhibitor of metalloproteinases-1 (TIMP-1) and aminoterminal propeptide of procollagen type III (PIIINP), has been recently proposed for fibrosis detection [16], [17]. A simplified version of this panel – called enhanced liver fibrosis (ELF) score- was shown to accurately predict significant liver fibrosis in different liver diseases [11], [18]–[21].

Extracellular matrix components may not only mirror the extent of liver fibrosis but are also involved in inflammatory processes. For instance, direct immunological impact of HA by regulating inflammatory cell recruitment and release of inflammatory cytokines has been described [22]. Vice versa, a variety of cytokines play a role in activating hepatic stellate cells for extracellular matrix production [23]–[25]. Moreover, hepatocyte apoptosis, which plays a role in inflammatory liver injury, has been mechanistically linked to stellate cell activation and increased fibrogenesis [26]. Activated hepatic stellate cells not only regulate fibrosis by secretion of extracellular matrix components but also induce an inflammatory response by expression of pro-inflammatory cytokines and receptors [27], [28]. Thus, multiple pathways of interaction between extracellular matrix production and inflammatory responses exist.

In the present study we have evaluated the performance of the ELF test against transient elastography for non-invasive assessment of fibrosis in a prospective biopsy-controlled manner. In this context we have analyzed the influence of possible confounders, such as liver inflammation or steatosis, on fibrosis detection by ELF score and transient elastography.

## Methods

### Patient Characteristics and Analysis of Liver Fibrosis

We investigated sera from 102 patients (52% male, age 18–75 years, mean 46.6±1.3 years) with chronic liver diseases (viral hepatitis, n = 55; autoimmune hepatitis, n = 7; Wilson’s diseases, n = 4; non-alcoholic fatty liver disease, n = 22; unknown origin, n = 14). Serum samples were analyzed for markers of the ELF score, including tissue inhibitor of matrix metalloproteinase 1 (TIMP-1), hyaluronic acid (HA), and amino-terminal propeptide of type III collagen (PIIINP). The proprietary assays developed for the ELF test by Siemens Healthcare Diagnostics Inc. (Tarrytown, New York, USA) were used and analyses were performed on an Immuno-1 auto-analyser (Siemens Healthcare Diagnostics Inc., Tarrytown, New York, USA). Results were entered into the established algorithm and expressed as score as described.^16^ In addition to the ELF score alanine and aspartate aminotransferase (ALT, AST) levels were determined. At the time of blood withdrawal, all patients obtained liver biopsy and liver stiffness measurement using the Fibroscan (Echosens, Paris, France). The fibrosis stage (F1–F6) was determined according to Ishak *et al.* [29]. The percentage of liver steatosis was assessed by the same pathologist. Patients were divided in low (F0-1; n = 68), moderate (F2-4; n = 23) and severe fibrosis (F5-6; n = 11). Demographic and clinical features of the patients are shown in **Table 1**. No significant differences in ALT levels, percentage of steatosis and body mass index (BMI) were observed between the different fibrosis groups. All liver stiffness (LS) measurements were performed by a single experienced investigator (M.D.) as described [8]. The result of liver stiffness determination was expressed in kPa and was the median of at least 10 individual measurements with a success rate of >60%. Valid LS values were obtained for all patients included in this study. Written consent was obtained from the patients participating in this study, and the consent procedure and study were approved by the Ethics Committee of Hannover Medical School.

**Table 1:** Demographic and clinical features of patients with different stages of fibrosis.

ISHAK	F0-1	F2-4	F5-6
Patient No.	68	23	11
Mean age ± SEM	45.6±1.8	48.2±2.0	49.1±3.3
Sex (% male)	51.5	52.2	54.5
Steatosis (%)	17.9±3.1	23.7±4.6	14.3±5.3
ALT (U/L)	67.9±5.8	80.7±8.7	92.7±19.5
BMI (kg/m^2^)	24.8±0.4	27.0±0.9	24.2±1.0
Biopsy Length (mm)	23.3±1.5	18.9±2.1	22.1±2.2

BMI, body mass index; SEM, standard error of the mean.

### Statistical Analyses

Statistical analyses were performed by using Graphpad Prism 5.0 and SPSS 19.0 software and confirmed by a professional statistician. Data are presented as box plot and whiskers analysis as well as mean ± standard error of the mean (SEM). The results obtained with the different serum markers or liver stiffness measurements were compared using the Mann-Whitney’s U test. Regression analyses were performed to calculate the Spearman rank correlation coefficient. Receiver operating characteristics (ROC) analysis was calculated. A P value <0.05 was considered significant. A multivariate logistic regression analysis was performed in order to adjust for variables found to be associated with fibrosis.

## Results

### Non-Invasive Assessment of Fibrosis Stages in Chronic Liver Diseases by ELF Test and Transient Elastography

**Figure 1:**
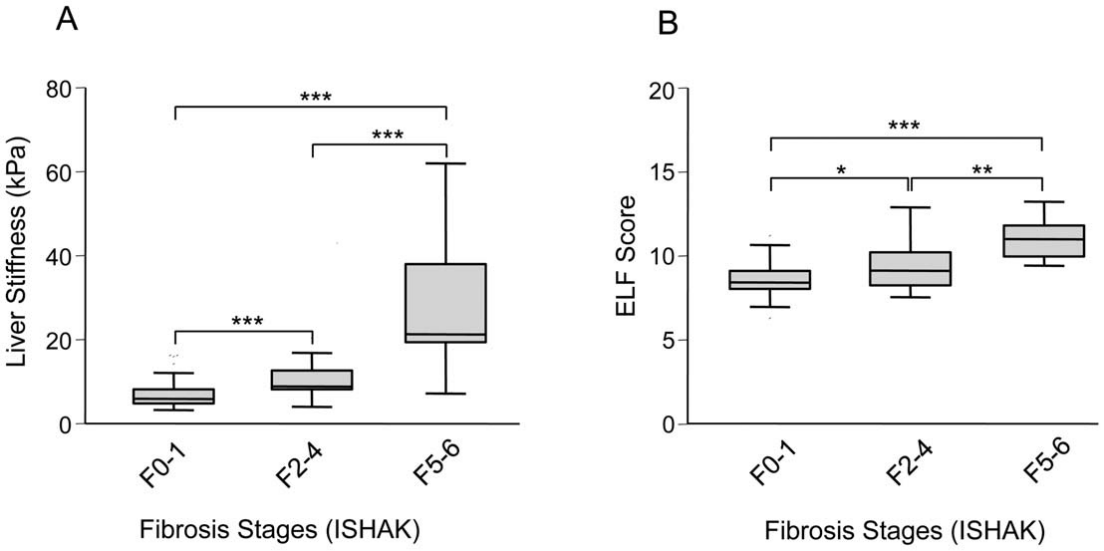
Measurement of liver stiffness by transient elastography and serological detection of ELF score in patients with chronic liver diseases and minimal (F0-1), moderate (F2-4) or high (F5-6) stages of fibrosis. Data are presented as box plots including medians and 25^th^ and 75^th^ percentiles. Both non-invasive methods can significantly discriminate between the different fibrosis stages. Transient elastography (A) allowed a better discrimination between minimal and moderate fibrosis stages (p<0.01) compared to ELF score (B; p<0.05). *P<0.05; **P<0.01; ***P<0.001. ELF, enhanced liver fibrosis.

The ELF test was compared with transient elastography for detection of different fibrosis stages in patients with chronic liver diseases (n = 102). Transient elastography allowed a better discrimination between low (F0-1; mean liver stiffness (LS) 6.9±0.4 kPa) and moderate (F2-4; mean LS 11.7±1.6 kPa) and between moderate and high fibrosis stages (F5-6; mean LS 27.3±4.8; **Figure 1A**) compared to the ELF score (F0-1: mean 8.6±0.1; F2-4: mean 9.3±0.3 and F5-6∶11.0±0.3; **Figure 1B**). Although both noninvasive methods could significantly discriminate between the different fibrosis stages, transient elastography revealed a higher significance (p<0.001) to distinguish between low and moderate fibrosis stages compared to the ELF score (p<0.05). Accordingly, the ELF score showed a broad overlapping range of F0-1 (6.3–11.2) and F2-4 (7.6–12.9) which was not observed for transient elastography (F0-1∶3.2–16.3 kPa and F2-4∶4.0–43 kPa). In line with this observation, regression analyses showed a significant correlation between transient elastography or ELF score and ISHAK fibrosis stages as well as between transient elastography and ELF score (**Table 2**).

**Table 2:** Correlation of ELF score or liver stiffness measured by transient elastograpgy with histological fibrosis.

	ELF Score	Liver Stiffness	ISHAK F
ELF Score		r = 0.479**	r = 0.525**
Liver Stiffness	r = 0.479**		r = 0.587**
ISHAK F	r = 0.525**	r = 0.587**	

** Correlation is significant at the 0.01 level (2-tailed). ELF, enhanced liver fibrosis.

### Predictive Value of the ELF Score and Transient Elastography to Detect Clinically Relevant or Progressed Fibrosis Stages

**Figure 2:**
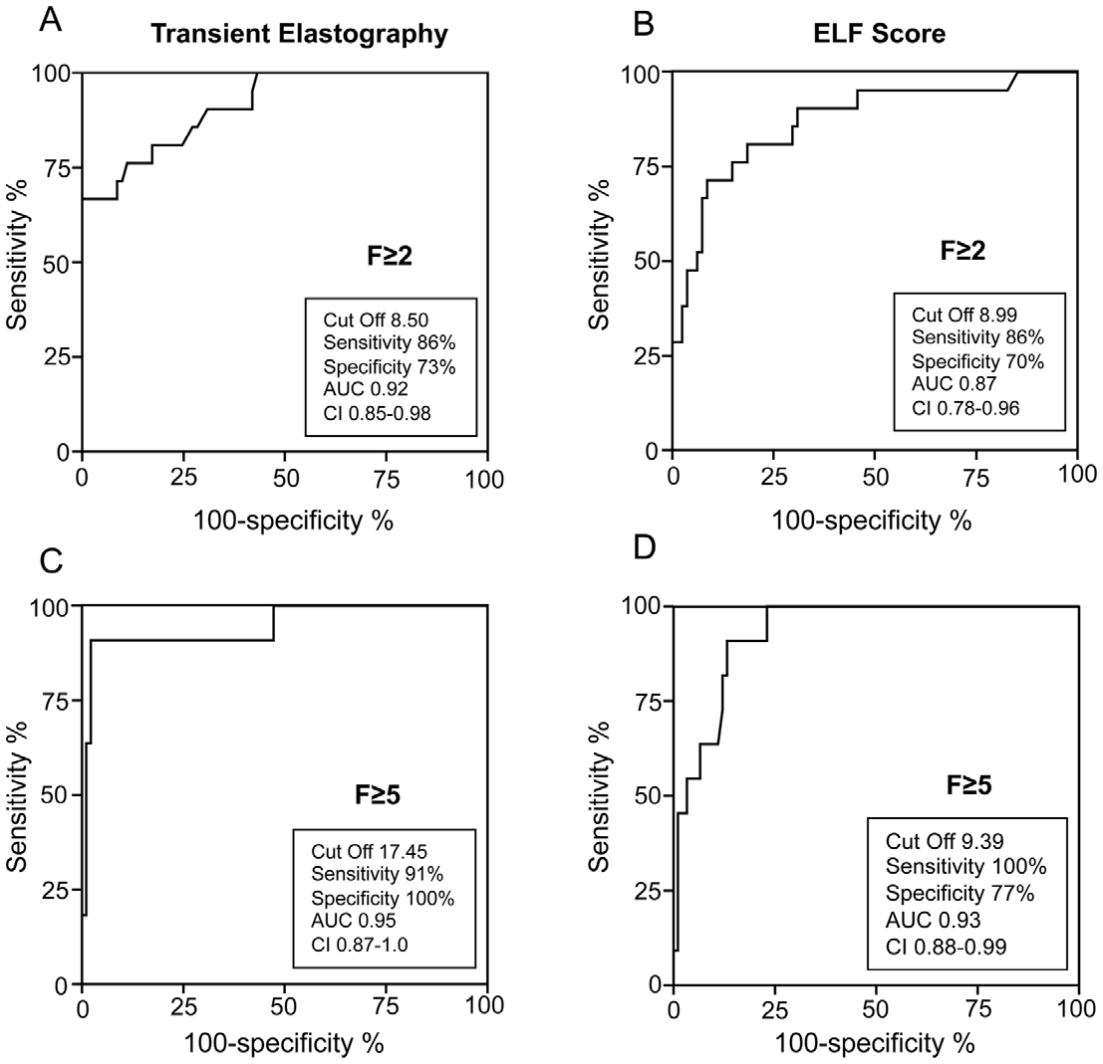
Prediction of relevant or advanced fibrosis stages by transient elastography and ELF score. The cut-off values of transient elastography (A, C) and ELF score (B, D) to predict fibrosis stages ≥F2 (A, B) or ≥F5 (C, D) with best compromise sensitivity/specificity were determined by ROC plot analysis. AUC, area under the curve; ELF, enhanced liver fibrosis; ROC, receiver operating characteristics.

We then calculated the cut-off values of the ELF Score and transient elastography to correctly predict clinically relevant stages of fibrosis (≥F2) or progressed fibrosis/cirrhosis (≥F5) with the best compromise sensitivity/specificity. To this end, we performed a ROC plot analysis including all patients (n = 102) with different fibrosis stages. The cut-off value of transient elastography of 8.5 kPa correctly predicted fibrosis stages of ≥F2 with a sensitivity of 86% and a specificity of 73% (AUC 0.92, confidence interval (CI) 95%: 0.85–0.98; **Figure 2A**). Similar results were obtained for the ELF score with a cut-off value of 8.99 that predicts clinically relevant fibrosis stages with a sensitivity of 86% and a specificity of 70% (AUC 0.87; CI95%: 0.78–0.96; **Figure 2B**). Compared to transient elastography that revealed with a cut-off value of 17.45 kPa a sensitivity of 91% and a specificity of 100% (AUC 0.95; CI95%: 0.87–1.0) for prediction of fibrosis stages of ≥F5 (**Figure 2C**), the ELF score showed a cut-off value (9.39) with higher sensitivity (100%) but lower specificity (77%; AUC 0.93; CI95%: 0.88–0.99; **Figure 2D**) for detection of progressed fibrosis/cirrhosis. However, the cut-off value of the ELF score to predict ≥F2 (8.99) was close to the cut-off value to predict ≥F5 (9.39). In contrast, the cut-off values of transient elastography for prediction of ≥F2 or ≥F5 fibrosis stages showed higher differences.

### Influence of Liver Inflammation on Transient Elastography and ELF Score

**Figure 3:**
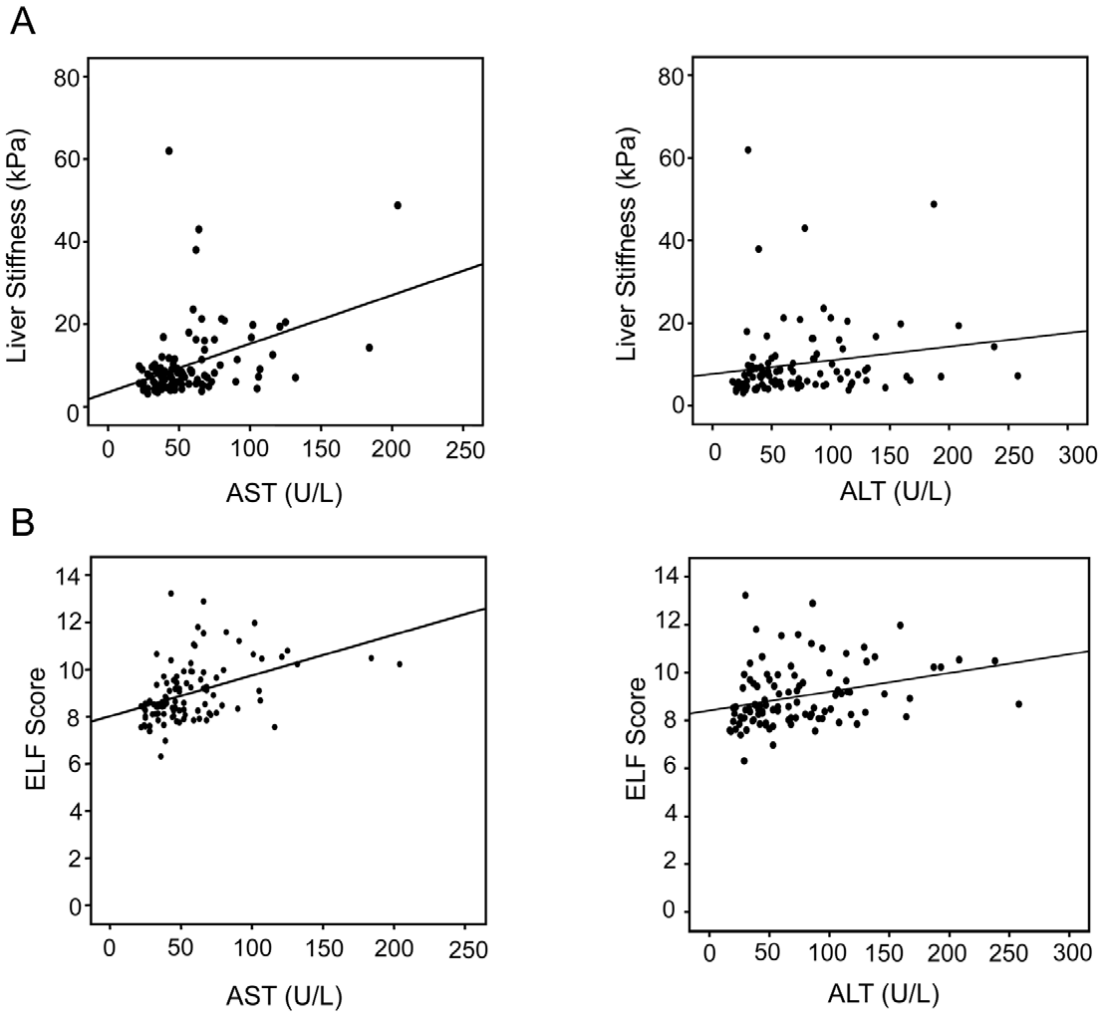
Regression analyses correlating liver stiffness measured by transient elastography or the ELF score with AST or ALT levels. A significant correlation (at 0.01/two tailed) was observed between liver stiffness (A) and AST or ALT levels as well as between ELF score (B) and AST or ALT levels. ALT, alanine aminotransferase; AST, aspartate aminotransferase; ELF, enhanced liver fibrosis.

To analyze a potential influence of liver inflammation on ELF score and transient elastography, we performed regression analyses comparing ALT or AST levels with ELF score and transient elastography. Both methods of fibrosis detection significantly correlated with AST and ALT levels (**Figure 3**). The ELF score showed a higher correlation with aminotransferase levels compared to transient elastography (**Table 3**). Similarly, the ELF score revealed a significantly higher correlation with inflammatory liver injury (ISHAK A-D) compared to transient elastography (**Table 3**). Thus, these data imply that the ELF score is more strongly influenced by inflammatory disease activity compared to transient elastography. In contrast to the ELF score, liver stiffness showed a weak but significant correlation with the percentage of liver steatosis (**Table 3**). To analyze the influence of inflammation or steatosis on prediction of relevant (≥F2) or progressed (≥F5) fibrosis stages, we performed a multivariate logistic regression analysis. This analysis showed that neither transient elastography nor the ELF score were significantly influenced by steatosis or inflammation (ISHAK A-D or ALT levels) in prediction of relevant or progressed fibrosis stages.

**Table 3:** Correlation of ELF score or liver stiffness measured by transient elastography with histological disease activity (ISHAK A-D), steatosis and aminotransferase levels.

	ELF Score	Liver Stiffness
AST	r = 0.475**	r = 0.431**
ALT	r = 0.362**	r = 0.297**
Steatosis %	r = 0.010	r = 0.257**
ISHAK A-D	r = 0.417**	r = 0.212*

Correlation is significant at the **0.01 or *0.05 level (2-tailed).

ELF, enhanced liver fibrosis.

## Discussion

Chronic liver diseases represent a substantial public health problem with a worldwide mortality of around 800.000 deaths per year [30]. A common pathological feature of chronic liver disease is fibrosis which is characterized by the progressive development of collagen-rich extracellular matrix and decreased matrix degradation due to an increase of inhibitors of matrix degradating enzymes, e.g. TIMPs [31]. Progression of liver fibrosis can result in liver cirrhosis with clinical complications due to loss of liver function and portal hytertension. Advanced fibrosis/cirrhosis are also considered a pre-cancerous state that provides a microenvironment which allows for the development of hepatocellular carcinoma. Thus, screening for fibrosis progression with non-invasive methods in everyday general practice is required to identify patients with increased risk of developing liver cirrhosis and associated complications. Vice versa, there is increasing evidence indicating that successful treatment of various chronic liver diseases is associated with fibrosis regression [32]. Moreover, novel antifibrotic agents targeting different factors of fibrogenesis revealed promissing results in animal models [33]. Monitoring of fibrosis regression during therapy of chronic liver diseases might be therefore also important for evaluation of treatment efficacy.

Ideally, non-invasive markers of liver fibrosis should be liver-specific and easy to perform with high diagnostic performance (compromise sensitivity/specificity) for accurate fibrosis staging. Among the most studied non-invasive detection methods of liver fibrosis is transient elastography. A prospective study in patients with chronic liver diseases demonstrated that measurement of liver stiffness by transient elastography is a reliable method to predict moderate or severe fibrosis stages, but shows less accuracay to differentiate between lower fibrosis stages according to METAVIR [7], [34], which was in line with observations of other studies [35]–[37]. In our prospective study of patients with chronic liver diseases, transient elastography was able to significantly (p<0.01) discriminate not only between moderate and high but also between low and moderate fibrosis stages according to Ishak classification [29]. Differences in the applied fibrosis scores as well as interobserver variability might account for the lower overlapping range between minimal and moderate fibrosis stages observed in the present compared to the latter studies. Indeed, interobserver agreement for transient elastography was found to be significantly reduced in patients with lower degrees of hepatic fibrosis [38], [39].

Compared to transient elastography, the ELF score revealed a lower significance (p<0.05) for discrimination between low and moderate fibrosis stages and showed a broad overlapping range for those stages. Nevertheless, AUC values for prediction of relevant fibrosis (≥F2) are high for both non-invasive methods with similar sensitivity and specificity. Both transient elastopgraphy and ELF score showed also a comparable high diagnostic accuracy to predict progressed fibrosis/cirrhosis (≥F5). The cut off-value of transient elastography for prediction of progressed fibrosis evaluated in this study was nearly the same (17.5 kPa) compared to that (17.6 kPa) of a previous study [7]. However, transient elastography showed a lower sensitivity for detection of fibrosis ≥F5 compared to the ELF score.

A recent study showed lower diagnostic performance for transient elastography in detection of liver cirrhosis compared to lower fibrosis stages [40]. One explanation for this observation could be that liver stiffness measurement topographically reflects liver architecture which is characterized by fibrotic septa and regenerative nodules in cirrhosis. Moreover, the architecture of liver cirrhosis shows differences between various liver diseases which might influence the sensitivity of cirrhosis detection by transient elastography. In contrast to transient elastography, the ELF score showed a lower specificity to predict progressed fibrosis. In this context it is interesting to note that the ELF score showed a higher correlation with ALT levels and with histological inflammatory liver injury compared to transient elastography. Thus, inflammatory disease activity might account for the lower specificity to detect progressed fibrosis by the ELF score. This might also be the reason for the lower performance of the ELF score compared to transient elastography in prediction of advanced fibrosis which has been recently demonstrated in patients with chronic hepatitis B [41].

There is also increasing evidence that liver stiffness is influenced by acute exacerbation of liver disease with ALT flares resulting in overestimation, e.g. up to three fold increase, of liver stiffness values [42]–[44]. Nevertheless, in acute liver failure it has been observed that liver stiffness correlates with tissue repair, e.g. markers of fibrogenesis [45]. However, we found a weaker correlation of transient elastography with ALT values and histological disease activity compared to the ELF score. This might be explained by the lack of disease flares with only moderately increased aminotransferase levels in the present study. In line with this observation, liver stiffness was not correlated with histological activity in chronic hepatitis C virus-infected patients that usually do not show ALT flares [35], [36]. Instead, we found a weak but significant correlation of transient elastography with steatosis which was not observed with the ELF test.

It has been reported that liver stiffness values are higher in subjects with enhanced BMI or metabolic syndrome [39], [46]. A multivariate analysis in patients with alcoholic liver disease showed a significant influence of steatosis on liver stiffness measurement [47]. In contrast, other studies did not reveal an influence of steatosis on fibrosis stage assessment by transient elastography [8], [36], [37]. Further studies are therefore needed to evaluate the influence of different grades of steatosis on liver stiffness measurements. A recently performed study comparing ultrasound-based methods with ELF score appeared confirming the diagnostic accuracy of those non-invasive methods for prediction of relevant fibrosis or cirrhosis [48]. However, the number of non-transplant patients with chronic liver diseases included in this study was lower (n = 59) compared to our study (n = 102), and unfortunately no information about possible variables that might influence fibrosis such as inflammation or steatosis was provided. Furthermore, the discriminative power of both methods for lower fibrosis stages, which is often relevant for clinical decision-making, remains unclear in this report.

Our present large biopsy-controlled prospective study showed that the ELF score reveals similar diagnostic accuracy to predict relevant (≥F2) or advanced (≥F5) stages of fibrosis compared with transient elastography. However, the cut-off values of the ELF score to predict relevant stages of fibrosis are close to the cut-off value for detection of progressed fibrosis whereas the respective cut-off values for transient elastography showed a higher difference. The ELF score appears less discriminative in lower fibrosis stages compared to transient elastography. Furthermore, the ELF score showed a higher correlation with inflammatory liver injury compared to transient elastography. These observations should be considered when making clinical interpretations or decisions on the base of ELF score values.
